# Non-Dissecting Distal Aortic and Peripheral Arterial Aneurysms in Patients With Marfan Syndrome

**DOI:** 10.3389/fcvm.2022.827357

**Published:** 2022-03-11

**Authors:** Quentin Pellenc, Auréline Boitet, Arnaud Roussel, Olivier Milleron, Pierre Mordant, Jean Senemaud, Pierre Cerceau, Guillaume Jondeau, Yves Castier

**Affiliations:** ^1^Department of Vascular and Thoracic Surgery, Bichat Hospital, Assistance Publique–Hopitaux de Paris (AP-HP), Paris, France; ^2^Centre de Référence pour le Syndrome de Marfan et apparentés, Bichat Hospital, Assistance Publique–Hopitaux de Paris (AP-HP), Paris, France; ^3^INSERM U 1148, LVTS, Bichat Hospital, Paris, France; ^4^Université de Paris, Paris, France; ^5^Department of Cardiology, Bichat Hospital, Assistance Publique–Hopitaux de Paris (AP-HP), Paris, France

**Keywords:** Marfan syndrome, aortic aneurysm, aortic dissection, peripheral aneurysms, vascular surgery

## Abstract

**Background:**

In Marfan syndrome (MFS), an aortic or peripheral arterial dilatation is usually the consequence of aortic dissection. Non-dissecting distal aortic and peripheral aneurysms (DAPA) are barely described. We sought to determine the incidence and prognostic impact of non-dissecting DAPA, requiring a surgical repair in a large population of patients with MFS.

**Methods:**

The patients referred to the French MFS reference center were included in a prospective database, and the patients treated for a non-dissecting DAPA between 2013 and 2020 were retrospectively reviewed. The first-line therapy was open surgery. The patients unfit for open repair or experiencing life-threatening complications underwent endovascular repair.

**Results:**

Among 1,575 patients with MFS, 19 (1.2%) were operated for 25 non-dissecting DAPA. The mean age was 42.4 ± 11.5 years. Non-dissecting DAPA involved the subclavian or axillary artery (*n* = 12), the descending or thoracoabdominal aorta (*n* = 6), the abdominal aorta andiliac arteries (*n* = 6), and the popliteal artery (*n* = 1). Open and endovascular repairs were performed in 22 and three cases, respectively. After a median follow-up of 54.2 months, no local recurrence was noticed and no secondary procedure was performed. Eight patients presented a new aortic event, including two aortic dissections and seven new aortic surgeries. Compared to the overall MFS population, the non-dissecting DAPA group presented a significantly higher risk of an aortic event (100 vs. 28%, *p* < 0.0001), a higher risk of aortic dissection (53 vs. 8%, *p* < 0.0001), and a higher rate of pejorative genetic mutations (68 vs. 40%, *p* = 0.011).

**Conclusion:**

Among the patients with MFS, the diagnosis of non-dissecting DAPA is infrequent but is associated with a significant adverse outcome, thus, advocating for a specific follow-up.

## Highlights

- Non-dissecting arterial aneurysms are infrequent in Marfan syndrome.
- Open and endovascular repair provided efficient et durable results.
- A significantly higher rate of aortic events occurred, compared to the Marfan population.
- This specific population requires a particular screening and follow-up.

## Introduction

Marfan syndrome (MFS) is the most common inherited connective tissue disorder involving the aorta. It is caused by different pathogenic variants in the fibrillin-1 gene (*FBN1*). Without intervention, the patients with MFS have an average life expectancy of 40 years, and 80% of mortality is directly related to aortic dilatation, dissection, and rupture (1). Effective therapy requires an early diagnosis and preventive measures, including a beta-blocker therapy and preventive surgery of ascending aorta (2). Current strategies to assess the future risk of aortic dissection or rupture are based primarily on monitoring the aortic diameter. However, many patients experienced a dissection or rupture below current intervention thresholds, urging the need to improve risk assessment of patients with MFS-associated aortopathy (1).

It is usually felt that only the aorta is affected in Marfan syndrome with an *FBN1* mutation, or that, if other arteries dilate, this is usually the consequence of aortic dissection. However, this is not true as fibrillin-1 is an ubiquitous protein, but the clinical consequences of extra-aortic alterations are rare in these patients, and not mentioned in European Society of Cardiology (ESC) Guidelines (2, 3) or a recent review on Marfan syndrome (4). Consequently, the incidence, management, and prognostic impact of distal aortic and peripheral aneurysms (DAPA) that are not related to aortic dissection remain unknown in patients with MFS. A recent study has suggested that aortic branch aneurysms could be present in up to one-quarter of patients with MFS and might independently predict the need for aortic surgery (5). We sought to determine the incidence, management, and prognostic impact of non-dissecting distal aortic and peripheral aneurysms (DAPA), requiring a surgical repair in patients referred to the French MFS reference center, with a special focus on the prognostic impact of this condition on a subsequent aortic event and overall survival.

## Methods

### Study Design

We set a retrospective study of a prospective database, including all patients with MFS, referred to the national reference center for Marfan syndrome and related disorders (www.marfan.fr) between January 2013 and May 2020. Diagnosis of Marfan syndrome was established in accordance with Ghent 2 criteria (6), including genetic testing (presence of *FBN1* gene mutation). Marfan-like syndromes such as Loeys–Dietz syndrome or aneurysms related to *TGFBR1/TGFBR2* and *SMAD3* mutations were excluded from this study. The patients treated for non-dissecting DAPA accounted for the study group, while the other patients were considered as the overall MFS population.

### Diagnosis

All the patients underwent computed tomography angiography (CTA) of the entire aorta at least yearly. Aortic branches were all visible on those CTA. Additional imaging (CTA or Duplex-scan) of supra-aortic trunks and lower limb arteries was performed in case of symptoms or guided by another indication. Aneurysm was defined when the arterial diameter was > 150% of the non-aneurysmal artery. Aneurysms evolving from dissected arterial segments were excluded from this study. The term DAPA was first used by Yetman and colleagues in 2011 to describe non-dissecting aneurysms involving aorta (excepted ascending aorta or aortic arch) or peripheral aneurysms (7). In our study, aneurysms were classified into distal aortic aneurysms (including the descending thoracic aorta, thoracoabdominal aorta, visceral aorta, and infra-renal aorta) and peripheral arterial aneurysms (including supra-aortic trunks, axillar artery, iliac arteries, common femoral artery, superficial femoral artery, and popliteal artery).

### Surgical Management

The management of the patients with MFS was discussed during a multidisciplinary meeting, including cardiologists, cardiac surgeons, vascular surgeons, radiologists, and anesthesiologists. The commonly accepted threshold diameter for surgery has already been published (8): 60 mm for thoracic and thoracoabdominal aorta, 50 mm for infra-renal abdominal aorta, 30 mm for subclavian and iliac arteries, and 20 mm for popliteal and axillar arteries. Open surgical repair was the first-line option. Arterial cross-clamping used systematically surgical clamps with atraumatic removable inserts (Intrack® clamp, Vitalitec International Inc., Plymouth, MA, USA) to avoid iatrogenic arterial dissection. Optimal control of the arterial pressure was ensured during the arterial cross-clamping. All anastomoses were performed using a standardized technique of a Teflon felt-supported polypropylene suture line. Endovascular treatments were limited to aortic aneurysms in the patients that are unfit for an open surgical repair or an emergent procedure (rupture). Endovascular protocol aiming at limiting the aortic wall aggression has already been described (9) and included using devices with no bare proximal stent or barbs, limiting the oversizing in case of landing in the native aorta, and privileging landing in a previous surgical graft whenever possible.

### Statistical Analysis

Data are presented as means with standard deviation or medians with ranges as appropriate. Nominal data were analyzed with a chi-square test or Fisher exact test. Continuous variables were analyzed with a Mann–Whitney test as their distributions were found to be non-normal. Aortic events were defined as aortic surgery or acute aortic dissection. Log-rank test was used to compare aortic events occurrence. Statistical analysis was performed using Prism 9.00 (GraphpadSoftware, San Diego, CA).

## Results

### Study Group

Among the 1,575 patients with MFS and *FBN1* gene mutation referred to the French reference center during the study period, 19 (1.2%) patients were operated for 25 non-dissecting DAPA and accounted for the study group. Their demographic characteristics are summarized in Table 1. The vast majority were males; the mean age at first DAPA treatment was 42.4 ± 11.5 years. The ascending aorta was dilated in all the patients but one (*n* = 18, 95%); acute aortic dissection occurred in almost half of them (*n* = 9, 47%). The mean age at the first aortic event was 32.5 ± 13.9 years.

**Table 1:** Baseline characteristics of the study group (*n* = 19 patients).

**Population characteristics**	**Study group**
	**(*n* = 19 patients)**
**Male**	**16 (84%)**
**Age at first aneurysm surgery (years)**	**42.4** ±**11.5**
ASA score ≥ 3	3 (16%)
Hypertension	6 (32%)
Diabetes mellitus	1 (5%)
Smoker/former smoker	3 (16%)
Dyslipdemia	1 (5%)
Chronic heart failure	1 (5%)
BMI > 30	1 (5%)
**Previous scoliosis surgery**	**9 (47%)**
**Mitral valve prolapse**	**9 (47%)**
**Ascending aortic dilatation**	**18 (95%)**
**Age at first aortic event (years)**	**32.5 ±13.9**
**Acute aortic dissection** Type A Type B	**9 (47%)** 4 (21%) 5 (26%)
**Previous aortic surgery**	**15 (79%)**
**Nb of previous aortic procedure (median):**	1.5 [0–3]
Ascending aorta repair Valve sparing technique Bentall procedure	14 (74%) 8 (42%) 6 (32%)
Aortic Arch repair	4 (21%)
Thoracic or Thoraco-abdominal repair	5 (26%)
Abdominal repair	3 (16%)

*ASA, American Society of Anaesthesiology. Bold values are main results*.

### Diagnosis

**Figure 1:**
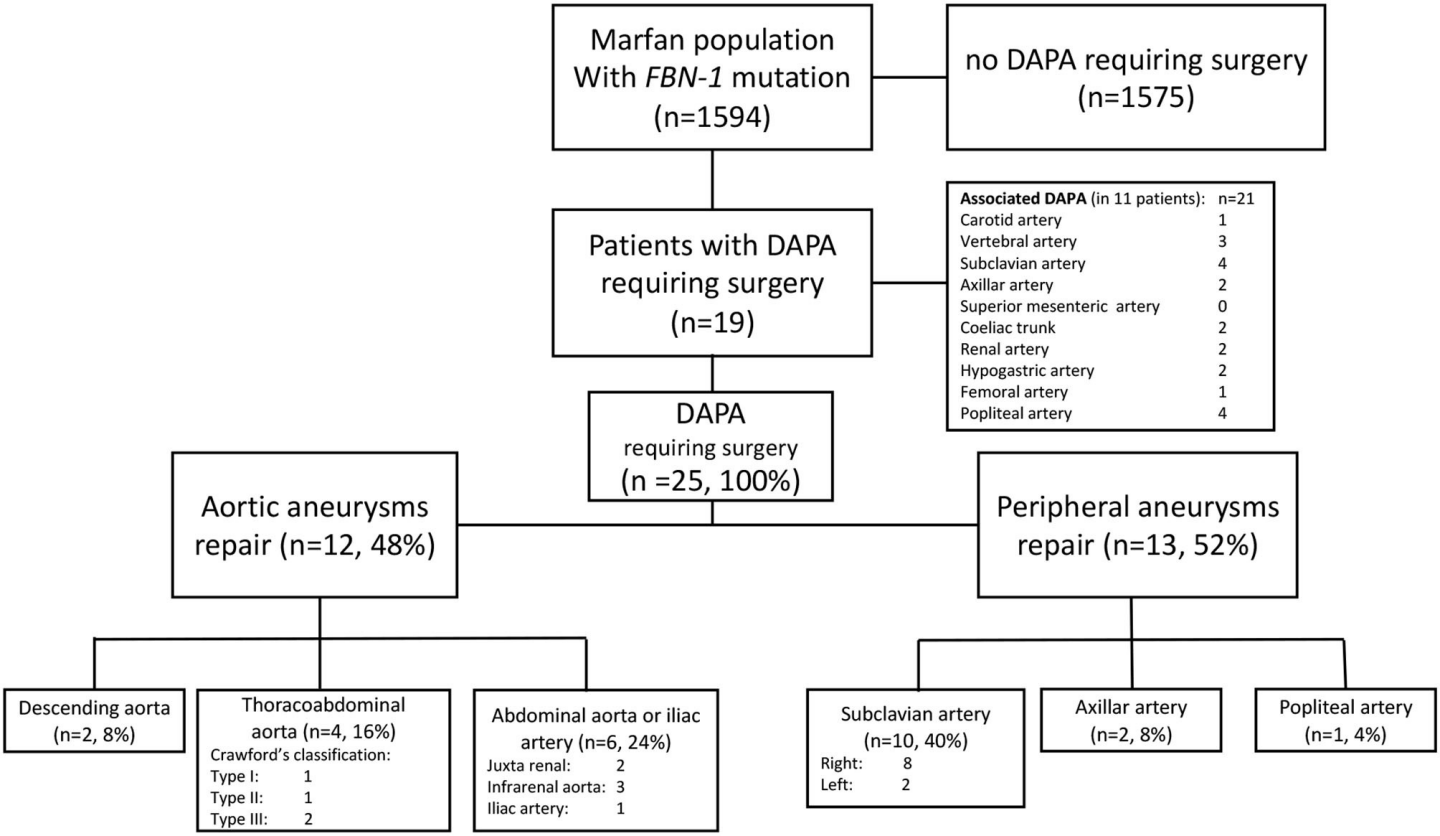
Characteristics of aneurysms in the study group (*n* = 25 aneurysms).

The characteristics of the 25 non-dissecting DAPA requiring surgery are reported in Figure 1. Twenty-one non-dissecting DAPA not requiring surgery were discovered in 11 patients of the study group, involving the internal carotid artery in 1, a subclavian artery in 4, the coeliac trunk in 2, the renal artery in 2, the hypogastric artery in 2, the popliteal artery in 4, the vertebral artery in 3, the axillar artery in 2, and the femoral artery in one case.

### Surgical Management

The surgical procedures are detailed in Table 2. An open repair was performed in 22 cases (88%). Treatment was performed in emergency for three patients: one for ruptured left subclavian aneurysm; one for a symptomatic type 3 thoracoabdominal aortic aneurysm (TAAA); and 1 for a symptomatic abdominal aortic aneurysm (AAA). A prosthetic graft was always used during open repairs. Endovascular repair was performed in three patients (12%): (i) a descending thoracic aortic aneurism below an open arch replacement and above a type IV open TAAA repair treated by thoracic endovascular aortic repair (TEVAR); (ii) a symptomatic type III TAAA in an obese patient treated by a branched aortic stent graft; and (iii) a ruptured distal left subclavian artery aneurysm treated with a covered stent.

**Table 2:** Surgical repair of aneurysms in the study group (*n* = 25 aneurysms).

**Surgical repair**	**Study group** **(*n* = 25 aneurysms)**
**Urgent repair** **Ruptured**	3 (12%) 1(4%)
**Open repair** Brachiocephalic trunk to subclavian artery bypass Ascending aortic graft-to subclavian artery bypass Left Carotid to subclavian artery bypass Axillar bypass Open DTAA open repair Open TAAA open repair Infra/juxta-renal aortic replacement (aorto bis-iliac) SFA transposition and graft bypass	22 (88%) 5 (20%) 2 (8%) 2 (8%) 2 (8%) 1 (4%) 3 (12%) 6 (24%) 1 (4%)
**Graft type:** Polyestar graft PTFE graft Veine bypass	20 (80%) 2 (8%) 0
**Endovascular repair** Branched endovascular aortic repair (TAAA) TEVAR (DTAA) Covered stent (subclavian artery)	3 (12%) 1 (4%) 1 (4%) 1 (4%)

*DTAA, descending thoracic aortic aneurysm; PTFE, polytetrafluoroethylene; TAAA, thoracoabdominal aortic repair; TEVAR, thoracic endovascular aortic repair*.

### Follow-Up

After a mean follow-up of 54 ± 29 months, all the patients were alive. The outcomes are summarized in Table 3. No redo surgery had been necessary on any non-dissecting DAPA. Primary and secondary patency rates were both 100%. No arterial dissection or anastomotic pseudoaneurysm was observed. Aneurysm shrinkage was observed in the three aneurysms that were endovascularly treated. Seven new aortic procedures were required in six patients (32%): open thoracoabdominal repairs after enlargement of the previous dissection in three patients, valve-sparing aortic root surgeries for aortic root dilatation in two patients, and TEVAR in two patients with previous surgery. At the end of the follow-up period, eight patients presented a new aortic event, including two acute type B aortic dissections in two patients and seven aortic surgeries in six patients.

**Table 3:** Follow-up of the study group (*n* = 19 patients and 25 aneurysms).

**Follow-up**	**Study group** **(n=19 patients /** **25 aneurysms)**
**Follow-up (months)**	**54.2** ±**29.3**
**Primary patency rate**	**25/25 (100%)**
**Patients with new aortic events during follow-up**	**8/19 (42%)**
**Patient requiring new aortic procedure** **New aortic procedures** Ascending aortic repair TEVAR Open TAAA repair	**6/19 (32%)** **7** 2 2 3
**Aortic dissection:** Type A Type B	**2/19 (10%)** 0 2

*TAAA, thoracoabdominal aortic aneurysm. Bold values are main results*.

### Prognostic Impact

**Figure 2:**
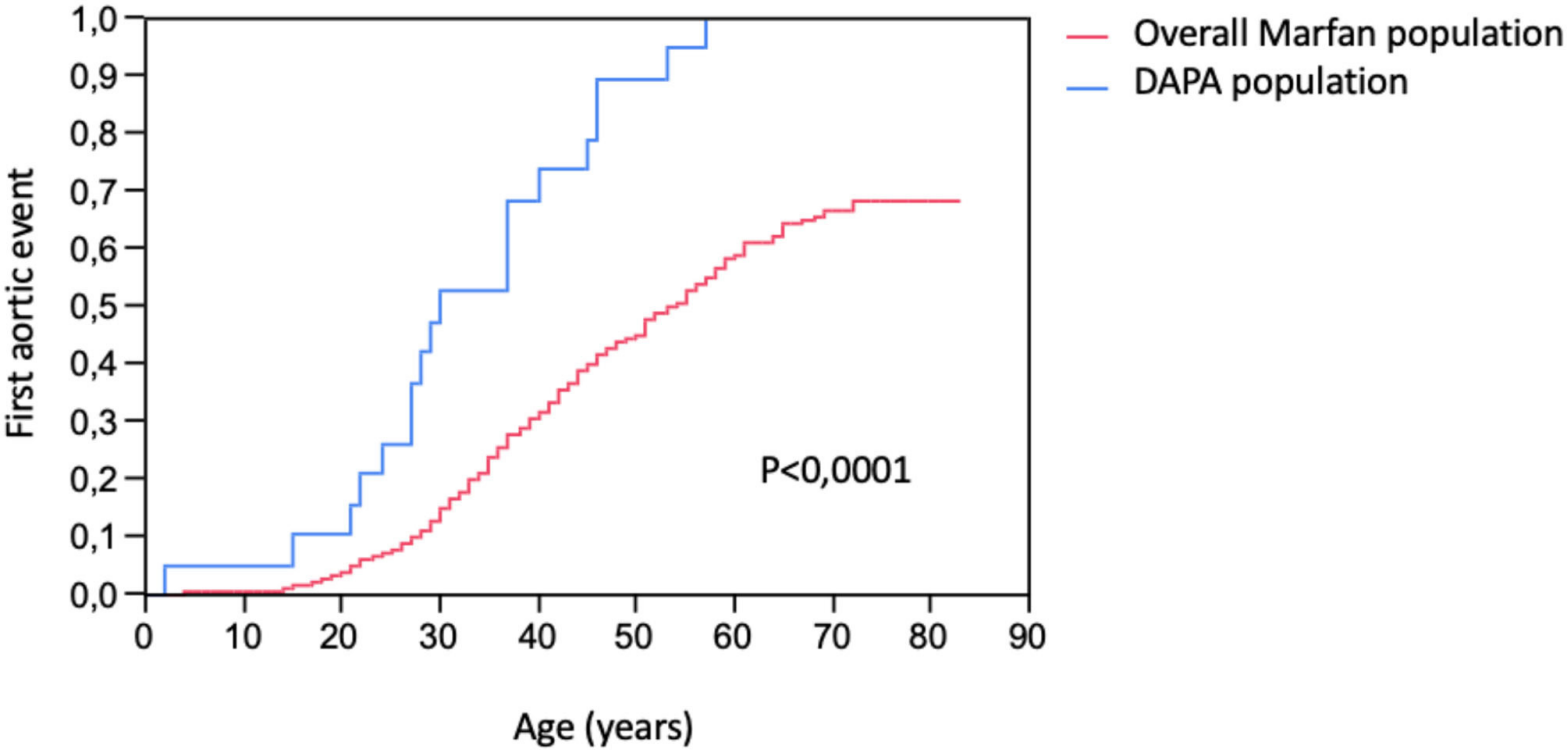
Kaplan–Meier estimation of first aortic events (ascending and distal aortic aneurysm surgery or aortic dissection) occurrence in DAPA (distal aortic and peripheral aneurysm) population and in the overall population of patients with Marfan syndrome with FBN-1 mutation.

In the study group, 10 patients (53%) presented an acute aortic dissection (4 type A and 7 type B, with one patient presenting two different dissections), and 12 patients (63%) required ascending aortic repair for aortic root dilatation. Compared to the overall MFS population, the study group presented a higher risk of an aortic event (100 vs. 28%, *p* < 0.0001) and, especially, a higher risk of aortic dissection (53 vs. 8%, *p* < 0.0001) as shown in Table 4 and Figure 2. The age at the first aortic event was significantly younger in the study group than in the overall MFS population (*p* < 0.0001). The FBN1 gene mutation characterization showed a significantly higher rate of premature termination codon (PTC) variants in the study group than in the overall MFS population (68 vs. 40%, *p* = 0.011).

**Table 4:** Comparison of the study group (*n* = 19) and overall population (*n* = 1,575).

	**Study group**	**Overall population**	***P*-value**
	**(*n* = 19)**	**(*n* = 1,575)**	
**Aortic event**	19 (100%)	436 (28%)	**<0.0001**
**Acute aortic dissection (A and B)**	10 (53%)	131 (8%)	**<0.0001**
**Mitral valve prolapse**	9 (47%)	594 (38%)	**0.387**
**Genetic analysis FBN-1 gene**			**0.011**
Premature termination codon	13 (68%)	627 (40%)	
In-frame variant	6 (32%)	948 (60%)	

*Aortic events included aortic dissection, ascending aorta repair, thoracoabdominal aorta repair. Bold values are main results*.

## Discussion

### Main Results Reminder

Using a large population of patients with MFS to study the incidence, management, and prognostic impact of non-dissecting distal aortic and peripheral aneurysms requiring surgery, we found an incidence of 1.2%, preferential localization on the distal aorta and subclavian arteries, safe surgical management through open repair, and an adverse impact on the aortic prognosis, advocating for an intensive follow-up of this specific subgroup of patients.

### Incidence and Localization

No recommendations exist concerning the peripheral aneurysms screening in patients with MFS (4, 10). This entity is, thus, probably underdiagnosed in a patient population with MFS, as suggested by Lopez–Sainz et al. who found the aortic branch aneurysms in almost 27% of the MFS-mutated population (5). In a preliminary report, Yetman et al. described that more than 30% (44/140) of their cohort of patients with MFS had distal aortic or peripheral non-dissecting aneurysms after thoracoabdominal CTA or MRI (7). Focusing on non-dissecting aneurysms requiring surgery, we found a lower but significant incidence of 1.2%.

### Surgical Management

Open surgery remains the first-line arterial surgical approach to patients with MFS. Vascular clamps applied to an MFS artery are likely to result in a different spectrum of arterial damage than in a non-MFS artery, advocating for the use of a traumatic vascular clamp (11). Wrapping of the anastomosis could also be used to prevent the apparition of anastomotic pseudoaneurysm as already described (12, 13). The use of Teflon-felt aortic suture lines provides a very low rate of anastomosis disruption (0.3% per year) with minimal risk of infection. However, due to patient general condition or an emergent setting, endovascular repair has been seldomly used to treat some patients with MFS in our study. Initially limited to bailout procedures in life-threatening situations, the use of TEVAR is slowly increasing in elective cases. The mitigated outcomes reported in older studies can probably be explained by the heterogeneity of surgical practices. In previous studies, the reintervention rates ranged from 14 to 60% mainly due to delayed failure of the technique and open conversion from 0 to 33% (14–22). However, the introduction of a dedicated approach using atraumatic stent-grafts, limited oversizing, and the graft in graft technique improves endovascular aortic repair results in the MFS population and brings the results to acceptable standards (9). In the present study, we strictly stuck to our specific approach for the two cases of aortic endovascular repair (1 off-the-shelve branched-EVAR and 1 TEVAR), which led to technical success and efficient mid-term aneurysm exclusion as previously reported (9).

### Prognostic Impact

Our study supports the idea that DAPA is a marker of increased aortic risk in the MFS population. In a recent study, Lopez–Sainz et al. demonstrated that aortic branch aneurysms are related to both age and ascending aorta dilatation. Their presence independently predicts the need for aortic surgery (the hazard ratio: 3.4; 95% confidence interval: 1.1 to 10.3; *p* = 0.028) (5). One explanation of the particular severity of this population could come from genetic analysis. Indeed, the FBN1 gene characterization showed a significantly higher rate of premature termination codon (PTC) variants in the DAPA population compared to in-frame variants (68 vs. 40%, *p* = 0.011). It is well-known that PTC variants lead to haploinsufficiency, and then, a decrease of fribrillin-1 protein expression (23). Patients with Marfan with PTC variants present more severe cardiovascular phenotypes than those with in-frame pathogenic variants (they being associated with dominant-negative effect) (23). In a recently published study from our group, the PTC variants experienced a significantly higher rate of ascending aorta surgery of acute aortic dissection (23).

### Clinical Implications

Our results are consistent with previous studies (5, 7, 24), showing an adverse impact of non-dissecting DAPA on the prognosis of patients with MFS, thus, highlighting the need for an adaptation of cardiovascular care with a specific follow-up protocol. These reports corroborate the need for screening distal aortic and peripheral aneurysms in patients with *FBN1*-mutated MFS, especially after the first aortic event (aortic surgery or acute aortic dissection). Similar to patients with mutations in the TGF-beta receptors, with a recognized risk of peripheral aneurysms (25), patients with MFS with an *FBN1* pathogenic variant should undergo screening with aortic CTA, associated with supra-aortic trunks CTA, and lower limbs CTA or Duplex-scan (26).

### Study Limitations

This study has several limitations. First, the studied sample of this uncommon presentation is small. This study is a retrospective analysis of prospectively collected data. The single-center recruitment means a referral bias remains possible, despite systematic familial screening and national recruitment through a national reference center.

## Conclusion

Non-dissecting distal aortic and peripheral aneurysms are an uncommon presentation of Marfan syndrome. The conventional open repair offers satisfactory long-term results. This subgroup of patients with MFS experiences increased the risks of dilatation of the ascending aorta, aortic dissection, and pejorative FBN-1 gene mutation, thus, advocating for complete arterial screening and specific follow-up. Further studies are needed to confirm the incidence and prognosis of non-dissecting distal aortic and peripheral aneurysms in patients with Marfan syndrome.

## Data Availability Statement

The raw data supporting the conclusions of this article will be made available by the authors, without undue reservation.

## Ethics Statement

The studies involving human participants were reviewed and approved by Commission Nationale de l'Informatique et des Libertés and Ethics Committee Comité de Protection des Personnes. The patients/participants provided their written informed consent to participate in this study.

## Author Contributions

QP, OM, and GJ conceptualized the study and designed the study. QP, AB, AR, and OM collected the clinical data. AR and JS performed data analysis. QP, OM, PM, JS, PC, YC, and GJ critically interpreted results. QP, AB, PM, and GJ drafted the manuscript. All the authors critically revised the manuscript and approved the submitted version of the manuscript.

## Conflict of Interest

The authors declare that the research was conducted in the absence of any commercial or financial relationships that could be construed as a potential conflict of interest.

## Publisher's Note

All claims expressed in this article are solely those of the authors and do not necessarily represent those of their affiliated organizations, or those of the publisher, the editors and the reviewers. Any product that may be evaluated in this article, or claim that may be made by its manufacturer, is not guaranteed or endorsed by the publisher.
